# Genetic Variation in the Psychiatric Risk Gene *CACNA1C* Modulates Reversal Learning Across Species

**DOI:** 10.1093/schbul/sby146

**Published:** 2018-10-10

**Authors:** Lucy Sykes, Josephine Haddon, Thomas M Lancaster, Arabella Sykes, Karima Azzouni, Niklas Ihssen, Anna L Moon, Tzu-Ching E Lin, David E Linden, Michael J Owen, Michael C O’Donovan, Trevor Humby, Lawrence S Wilkinson, Kerrie L Thomas, Jeremy Hall

**Affiliations:** 1 Neuroscience and Mental Health Research Institute, Cardiff University, Cardiff, UK; 2 School of Psychology, Cardiff University, Cardiff, UK; 3 Department of Psychology, Durham University, Durham, UK; 4 School of Medicine, MRC Centre for Neuropsychiatric Genetics and Genomics, Cardiff University, Cardiff, UK; 5 School of Biosciences, Cardiff University, Cardiff, UK

**Keywords:** reversal learning, rat, behavior, BDNF, translational, calcium

## Abstract

Genetic variation in *CACNA1C*, which encodes the alpha-1 subunit of Ca_v_1.2 L-type voltage-gated calcium channels (VGCCs), has been strongly linked to risk for psychiatric disorders including schizophrenia and bipolar disorder. How genetic variation in *CACNA1C* contributes to risk for these disorders is however not fully known. Both schizophrenia and bipolar disorder are associated with impairments in reversal learning (RL), which may contribute to symptoms seen in these conditions. We used a translational RL paradigm to investigate whether genetic variation in *CACNA1C* affects RL in both humans and transgenic rats. Associated changes in gene expression were explored using in situ hybridization and quantitative PCR in rats and the BRAINEAC online human database. Risk-associated genetic variation in *CACNA1C* in healthy human participants was associated with impairments in RL. Consistent with this finding, rats bearing a heterozygous deletion of *Cacna1c* were impaired in an analogous touchscreen RL task. We investigated the possible molecular mechanism underlying this impairment and found that *Cacna1c* +/− rats show decreased expression of *Bdnf* in prefrontal cortex. Examination of BRAINEAC data showed that human risk-associated genetic variation in *CACNA1C* is also associated with altered expression of brain-derived neurotrophic factor (*BDNF*) in the prefrontal cortex in humans. These results indicate that genetic variation in *CACNA1C* may contribute to risk for schizophrenia and bipolar disorder by impacting behavioral flexibility, potentially through altered regulation of *BDNF* expression in the prefrontal cortex. Tests of RL may be useful for translational studies and in the development of therapies targeting VGCCs.

## Introduction

Genetic variation in *CACNA1C*, which encodes the alpha-1 subunit of Ca_v_1.2 L-type voltage-gated calcium channels (VGCCs), has been strongly linked to risk for psychiatric disorders including schizophrenia and bipolar disorder.^1,2^ The association between common genetic variation in intron 3 of the *CACNA1C* gene and schizophrenia and bipolar disorder has been confirmed by multiple studies across different populations.^1,3–7^ Risk mediated through common variants in *CACNA1C* has also been found to be shared across disorders, with Smoller et al^2^ finding similar associations with attention deficit hyperactivity disorder (ADHD), autism spectrum disorder, bipolar disorder, major depressive disorder, and schizophrenia. Expression studies in cell lines and postmortem tissue have suggested that the intron 3 risk variants may act to decrease expression of *CACNA1C*, although the exact direction of effect has varied between studies and tissue types.^8–12^ Rare deleterious mutations in VGCCs have also been found to be associated with risk for schizophrenia.^13^ Notably, this enrichment was primarily driven by pore-forming alpha-1 subunits and alpha-2-delta auxiliary subunits of VGCCs, including deleterious mutation loss of function variants in *CACNA1C.*^13^

The robust association of *CACNA1C* with multiple psychiatric disorders has led to the investigation of potential endophenotypes and downstream biological mechanisms that may link genetic risk to disease-relevant behavior. Previous studies have identified a link between risk-associated variation in *CACNA1C* and a range of tasks requiring cognitive and behavioral flexibility. Specifically, genetic variation in *CACNA1C* and related VGCCs has been strongly associated with working memory performance as assessed by the N-back test, which is known to depend on prefrontal cortex (PFC) function.^14–16^ Genetic variation in *CACNA1C* has similarly been shown to affect logical memory in patients^17^ and verbal working memory in healthy controls.^18^*CACNA1C* genotype has also been found to affect reward learning.^19,20^ Genetic imaging studies have been conducted to identify neural circuits affected by genetic variation in *CACNA1C* and have shown altered activation of fronto-limbic brain circuitry in risk allele carriers.^12,15,19,21^

Reversal learning (RL) tasks represent a powerful method for investigating cognitive flexibility with considerable potential for cross-species translational studies.^22^ RL involves the inhibition of a previously learnt association between a stimulus and reward and the acquisition of a new opposite contingency or rule. It is a necessary form of behavioral flexibility that allows new experience to influence behavior.^23^ Deficits in this form of learning can lead to inappropriate perseverative behavior that is not supported by, or beneficial in, the current environment. Impairments in RL have been reliably observed in patients with a range of psychiatric conditions including schizophrenia and bipolar disorder.^24–29^ Furthermore, deficits in RL have also been observed in first-episode psychosis patients^30^ and in the context of high polygenic risk for schizophrenia,^31^ suggesting that RL deficits may reflect a causal mechanism linking common genetic schizophrenia risk to symptomology. Homologous fronto-limbic networks have been found to be involved in RL in both rodents and humans,^22,23,32^ and the development of a touchscreen-based RL paradigm for rodents makes the task highly translatable.^33,34^

At a molecular level, the neurotrophin brain-derived neurotrophic factor (BDNF) has been shown to play a critical role in PFC during RL.^35,36^ Mice lacking the main activity-regulated form of *Bdnf* show impaired plasticity in the PFC and behavioral deficits in RL tasks.^35,36^ Furthermore, manipulations in animal models that decrease prefrontal *Bdnf*, including maternal separation, have been shown to result in impairments in RL.^37,38^ VGCCs, including *CACNA1C*, have been shown to be central to the activity-dependent regulation of BDNF, suggesting the possibility that genetic variation in *CACNA1C* may affect cognitive flexibility in part through altering *Bdnf* expression in the PFC.^39–43^

On the basis of these findings, we sought to investigate the effect on RL of *CACNA1C* risk variants in humans and altered *Cacna1c* dosage in rats. We additionally assessed the impact of genetic variation in *CACNA1C* on prefrontal *BDNF* levels in both humans and rats. By conducting these behavioral, genetic, and molecular studies in parallel, we aimed to increase confidence in our findings and develop translational models for the future development of novel therapies for psychiatric disorders targeting VGCCs.

## Methods and Materials

### Human Participants

One hundred right-handed Caucasian healthy participants (aged 19–47 years) derived from the local community took part in the study. Ethical approval was given by the ethics committee of the School of Psychology, Cardiff University, and informed consent was obtained from each participant prior to the study. Participants had no history of psychiatric illness (themselves or first-degree relative) and did not report taking any psychotropic medication or illegal substances. All participants were right-handed, university graduates (with 17+ years’ education). A total of 84 participants (mean age 23.95 ± 3.64 SD, 49 women) were used for analysis, after exclusion for quality control of genetic data (*N* = 10) or incomplete/missing behavioral data (*N* = 6). The data used in this study have previously been used to observe associations between genome-wide polygenic risk for schizophrenia and RL behavior.^31^

### Probabilistic RL Behavioral Task

**Fig. 1.**
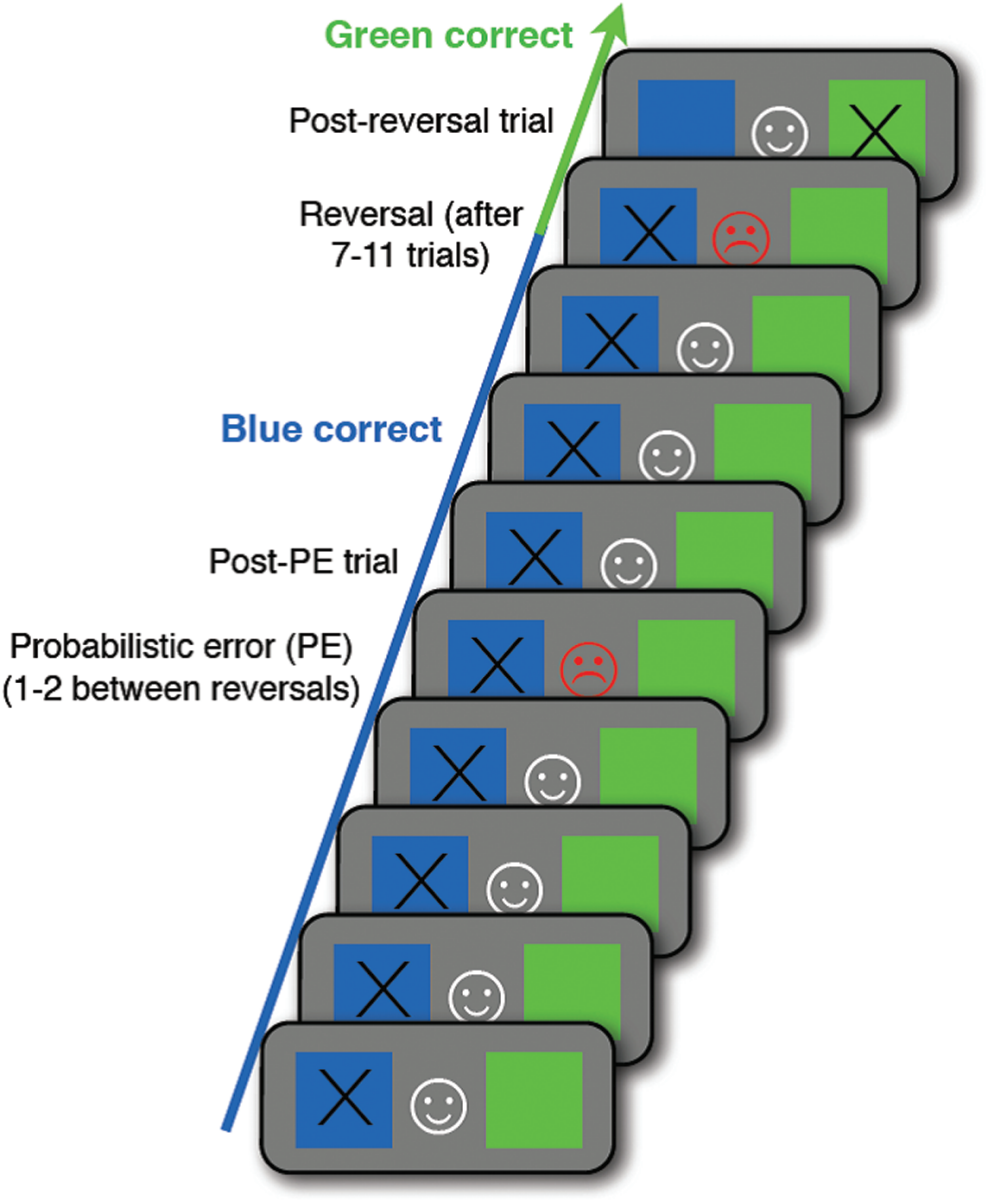
Reversal learning paradigm. Participants were presented with a choice between blue or green squares. Correct choices were rewarded with monetary winning and incorrect responses with a fine. One or two probabilistic error trials were included within each session block in which the “correct” response was punished.

Participants were asked to make a choice between a blue and a green stimulus presented simultaneously (figure 1). A correct response was followed by a white “smiley” face and rewarded with a +1 p monetary reward and a red “frowny” face and a −1 p monetary punishment followed an incorrect response. Participants were told to maximize their “earnings” throughout the task. After 7–11 trials, the contingencies were reversed and the previously rewarded stimulus was now punished. In total there were 12 reversal sessions (total of 108 choice trials). Each session included 1 or 2 “probabilistic errors” in which the correct response was punished to ensure sufficient difficulty in the task. “Total Earnings” were calculated for each participant for the full task (in pence) based on the amount of rewards and punishments they received throughout. Performance following the first contingency reversal (“Reversal Accuracy”) was calculated as percentage correct for that session block.

### DNA Extraction and Participant Genotyping

Samples of saliva were collected from participants for genotyping using Oragene DNA (OG-500) kits (DNA Genotek Inc). Genotyping was conducted using a custom HumanCoreExome BeadChip Kit (Illumina), which included 570 038 variants. Data were quality-controlled in PLINK.^44^ Participants were removed if data suggested non-European ancestry, relation to other participants, or incomplete genotyping <97%. Genotypes were then imputed by estimating haplotypes using SHAPEIT and imputing genotypes from the reference set 1000 genomes (December 2013, release 1000 genomes haplotypes phase 1 integrated variant set) using IMPUTE2^45,46^. For the current analysis, genotypes were extracted from the full single-nucleotide polymorphism (SNP) dataset for rs1006737 and rs2007044 only. These SNPs were selected because rs1006737 is the most consistently reported disease-associated SNP in *CACNA1C* across disorders,^47^ and the most recent schizophrenia genome-wide association study found rs2007044 to have the strongest association with schizophrenia (as well as confirming association with rs1006737).^48^ These 2 SNPs are in high linkage disequilibrium (*r*^*2*^ = .8). Allelic association analyses were conducted to determine whether incidence of each individual allele at each SNP location was associated with performance. Student’s t tests were used to compare performance for each allele.

### Animals

Eighty-eight male Sprague‑Dawley *Cacna1c* heterozygous (HET) knockout rats were housed with wild-type (WT) littermates in groups of 1–4. Heterozygous *Cacna1c* Sprague–Dawley rats were obtained from cryopreserved embryos, created with zinc-finger nuclease (ZFN) technology (Sage Research Labs). A pair of 5-finger ZFNs, recognizing a total of 30 base pairs, was used to target exon 6 (location 460649–460652 bp in genomic sequence), resulting in a frameshift and an early stop codon. Manipulation of founders was confirmed by PCR (Fwd: 5′- GCTGCTGAGCCTTTTATTGG-3′; Rev: 5′- CCTCCTGGATAGCTGCTGAC-3′) and sequencing. Details of basic molecular and behavioral characterization of *Cacna1c* HET rats are given in the Supplementary Material, including supplementary figure S1. Given the limitations of individual Ca_v_1.2 alpha-1 subunit antibodies, we used 2 separate antibodies in western blots and an ELISA assay to confirm protein levels are reduced in our heterozygous line (see Supplementary Material). All procedures were conducted in accordance with the guidelines published in the Institute of Laboratory Animals Resources Commission on Life Sciences 1996 *Guide for the Care and Use of Laboratory Animals.*

### Animal Touchscreen RL Paradigm

**Fig. 2.**
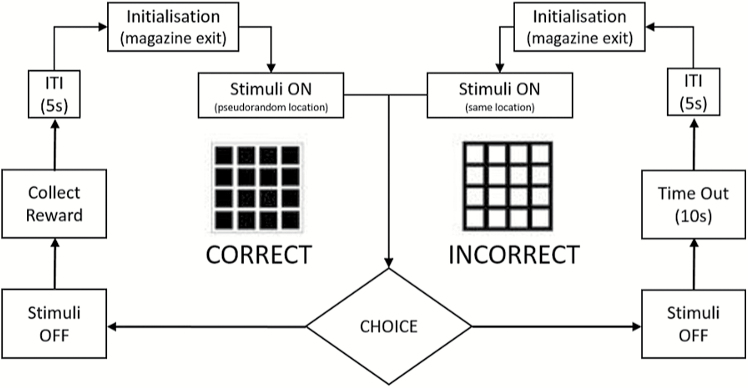
Schematic of trials used for Visual Discrimination and Reversal sessions for correct and incorrect responses. Inter trial interval (ITI).

In this study, 13 WT and 12 HET rats were tested using a touchscreen RL task.^33^ Animals were placed on water restriction on testing days to maximize motivation for reward, in accordance with Home Office regulations. Animals were run in sets of 4 using Bussey–Saksida rat touchscreen chambers (model 80604; Campden Instruments) with accompanying Animal Behavior Environment Test (ABET) II software (model 89505; Campden Instruments). The overall stages and criteria for moving on to the next phase of the experiment are shown in supplementary table S1. Animals were pretrained to collect the reward (10% sucrose solution) from the magazine (Habituation), and to nose poke a stimulus to receive reward (Must Touch). Following completion of pretraining, animals were presented with a pair of stimuli (S+ and S−, side of presentation was allocated pseudorandomly). Touches to the S+ resulted in delivery of the reward and the termination of the trial. Touches to the S− resulted in the termination of the trial and a 10 s time-out period before the next trial could be initiated (figure 2). Visual discrimination training continued until an animal was performing at 80% correct within a session on 2 consecutive days (supplementary table S1), following this the contingencies were reversed (S+ becomes the S−, and vice versa). Early phase reversal was characterized by overcoming the previously learnt association and obtaining at least 50% correct responding to the newly rewarded stimulus. Late phase reversal was defined as obtaining 80% correct responding to the newly rewarded stimulus on 2 consecutive days (supplementary table S1).

Correct responses and number of errors (incorrect responses) for each session were recorded to establish performance across phases and perseveration. For each session of discrimination and RL, % correct response [(correct responses/total responses) × 100] was calculated. The number of sessions and trials required to reach criterion were recorded for each condition. Animals were only included if they had reached criterion for that training phase. The proportion of animals of each genotype to complete each phase was compared to establish success rate. Latencies to respond to stimuli were recorded to indicate speed of decision and response. Latencies were averaged across the first 3 sessions and the last 3 sessions to give an indication of change within phase. Student’s t tests were used to compare the number of sessions taken to complete and error number per session and chi-squared tests were used to compare the frequency of completion for experimental condition by genotype. Repeated measures ANOVAs with time and genotype were conducted to analyze response latencies across sessions.

### Quantitative In Situ Hybridization

In situ hybridization was used to quantify the expression of *Bdnf* and *Cacna1c* in rodent tissue using established techniques.^49,50^ Full details of the in situ hybridization methods and analysis are given in the Supplementary Material.

### Quantitative PCR

Quantitative PCR (qPCR) was conducted with SensiMix SYBR Green (Bioline) as described in Supplementary Material.

### Human Gene Expression Analysis by Risk SNP

We examined the effects of genetic variation in *CACNA1C* at rs1006737 and rs2007044 on postmortem gene expression using the BRAINEAC online database (www.braineac.org).^51^ Using the BRAINEAC online general user interface, the expression of BDNF was stratified by rs1006737 and rs2007044 for each individual probe set available. Allele frequencies for the two SNPS were rs1006737: A = 29.1%, G = 70.9%; rs2007044: G = 36.9%, A = 63.1%.

## Results

### Healthy Human CACNA1C Risk Allele Carriers Show Altered RL

**Fig. 3.**
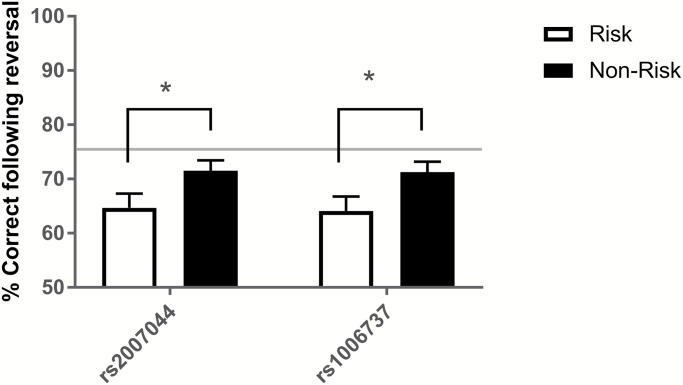
Comparison of accuracy following first reversal for risk and non-risk alleles at rs1006737 and rs2007044. Risk allele was associated with significantly reduced performance compared to non-risk allele carriers. Bars represent average % correct. Error bars are standard error of the mean (SEM). *P < .05.

The risk allele at both rs1006737 and rs2007044 was associated with fewer correct responses during RL (figure 3). Average performance associated with the risk allele (A) at rs1006737 was 64.08% (SEM = 2.66%) compared with 71.27% (SEM = 1.90) associated with the non-risk allele (G); and at rs2007044 average performance associated with the risk allele (G) was 64.66% (SEM = 2.41) compared with 71.69% for the non-risk allele (SEM = 2.04%). Student’s t tests between revealed a significant effect of risk allele at rs1006737 and rs2007044 with first “Reversal Accuracy” [t(166) = −2.242, *P* = .026 and t(165) = −2.230, *P* = .027, respectively]. There were no significant effects of risk allele at rs1006737 (t(166) = −1.562, *P* = .120) or rs2007044 on “Total Earnings” [t(165) = −1.122, *P* = .264] indicating comparative levels of task motivation. Further analysis confirmed that the association between *CACNA1C* risk allele and performance was specific to trials following reversal and was not affected by correction for relevant covariates including age and gender (see Supplementary Material including supplementary figure S2 and supplementary table S3).

### Cacna1c Heterozygote Rats Show Altered RL

**Fig. 4.**
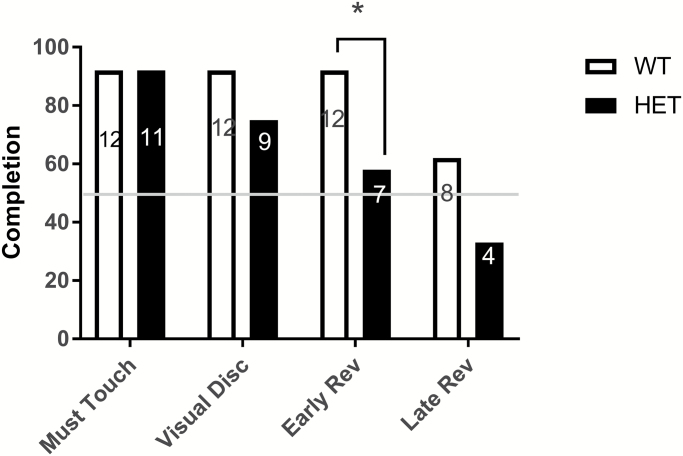
Completion rates (% of animals of each genotype) for each experimental condition from Must Touch to Reversal Criterion. Fewer HET animals compared to WT completed Early Reversal stage of 50% performance levels following reversal. *P < .05. WT = wild type.

There were no differences between rat genotypes in number of sessions to reach criteria for Habituation or Must Touch [t(21) = −0.249, *P* = .806 and t(18.206) = 1.323, *P* = .202, respectively]. Twelve of the 13 WT animals and 9 out of 12 HETs animals reached criteria for “Visual Discrimination” with no difference in the proportion of completion [χ(1) = 1.391, *P* = .238] or number of sessions taken to reach criteria [t(19) = −0.833, *P* = .415]. Analyses were conducted to compare the proportion of WT and HET animals that completed the “Early” reversal stage, indicating a successful inhibition of response to prior contingency. Fewer HETs completed “Early Reversal,” with 12 WT animals successfully completing early reversal (92%), compared with 7 HET animals (58%) [χ(1) = 3.949, *P* = .047] (figure 4). Mean number of sessions for the completion of each training session by HET and WT animals are shown in supplementary table S4. This pattern was reflected in the total number of errors made at each stage. Specifically, there was no difference between genotypes in total errors during the Visual Discrimination phase [t(21) = −0.001, *P* = 1], but HET animals made significantly more errors compared to WT during Early Reversal [t(17) = 2.176, *P* = .044].

Comparison of response latencies further indicates altered behavior in HET animals following reversal of contingencies. There was no evidence of a difference in time to respond to stimuli during Visual Discrimination [*F*(1,19) = 1.134, *P* = .300]; however, HET animals responded significantly faster than WT during early reversal [*F*(1,16) = 10.921, *P* = .004] (supplementary figure S3).

### Reduced Prefrontal Cortex Bdnf Expression in Cacna1c Heterozygote Rats

**Fig. 5.**
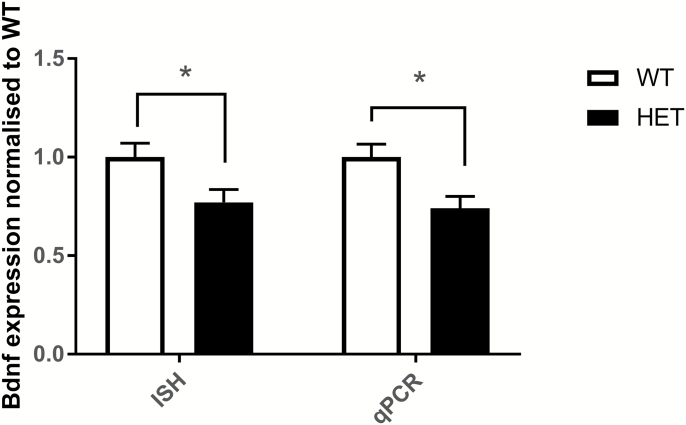
BDNF mRNA expression levels compared between WT and HET animals in PFC. Results show a 22% and a 26% reduction in PFC in HET animals with ISH and qPCR respectively. Bars represent mean expression normalized to WT. *P < .05. BDNF = brain-derived neurotrophic factor; ISH = in situ hybridization; PFC = prefrontal cortex; qPCR = quantitative PCR; WT = wild type.

qPCR analysis revealed a significant decrease in the expression of *Bdnf* in the PFC of *Cacna1c* heterozygous rats [t(19) = −2.223, *P* = .039] (figure 5), with no differences observed in the CA1 region of the hippocampus [t(19) = 0.741, *P* = .408]. In situ hybridization was used to further compare levels of *Bdnf* mRNA expression in the PFC of a separate cohort of *Cacna1c* HET (*n* = 5) and WT (*n* = 5) rats. Analysis confirmed that HET animals showed significantly reduced expression of total *Bdnf* mRNA levels in the PFC compared with WT animals [t(8) = 2.316, *P* = .049] (figure 5), with no difference in expression in the hippocampal CA1 field [t(8) = −1.418, *P* = .194].

***Risk SNPs in* CACNA1C *are associated with decreased expression of BDNF in human frontal cortex***

We investigated whether genetic variation at the two *CACNA1C* risk loci is associated with expression changes of *BDNF* in the human PFC. Examination of the BRAINEAC database revealed a significant association between genotype at rs1006737 and rs2007044 and the expression of *BDNF* (*P* = .007 and = .042, respectively) as assessed by probe set 3367273 (supplementary figure S4). Risk allele carriers at both SNPs showed significantly reduced expression of *BDNF*, with homozygous carriers having a more pronounced reduction.

## Discussion

### Summary

We report that healthy human subjects bearing alleles in the *CACNA1C* gene associated with risk for psychiatric disorders show poorer reversal performance on a probabilistic RL task than non-risk allele carriers. We also report convergent evidence that rats heterozygous for the *Cacna1c* gene are impaired in the same phase of RL. We show that *Cacna1c* hemizygosity in rats is associated with decreased *Bdnf* expression in the PFC, and that human risk variants in *CACNA1C* are also associated with decreased *BDNF* expression as indexed by selective markers in postmortem tissue. Overall, these results suggest that genetic variation in *CACNA1C* may contribute to risk for psychiatric disorders through impacting cognitive flexibility, and that at a molecular level, this may be in part caused by alterations in *BDNF* expression.

### Human Risk Alleles in CACNA1C Impact RL

We found that risk-associated common genetic variants in *CACNA1C* were found to be associated with deficits in the reversal stage of a probabilistic RL task. Specifically, in healthy human participants the risk-associated alleles of *CACNA1C* were associated with impaired performance in a probabilistic RL task, indicating an impact of genetic variation in *CACNA1C* on cognitive flexibility. These results suggest that the risk variant may be affecting neural circuitry involved in the comparison and updating of existing information to inform subsequent behavior and forming optimal choice contingencies. Prior studies in human participants suggest a specific role for the PFC, especially orbito-frontal regions, and the striatum in RL.^22^ Furthermore, human genetic imaging studies have indicated an impact of genetic variation of *CACNA1C* genotype on fronto-limbic brain activation and connectivity, although the impact of *CACNA1C* genotype on brain activation during RL has not been specifically investigated.^12,15,21,52^ Polygenic risk scores for schizophrenia, to which genetic variants in *CACNA1C* contribute, have however been shown to influence fronto-striatal brain activation during RL.^31^

Cognitive flexibility is known to be impaired in a range of psychiatric disorders that have been associated with genetic variation in *CACNA1C*. Deficits in RL have, for example, been demonstrated in patients with psychotic disorders including schizophrenia and bipolar disorder.^23,25–27,29,30^ In schizophrenia, impairments in RL have been shown to be related to clinical ratings of symptoms including disorganization and thought disorder, findings that were not explained by impairments in general intelligence.^26^ Furthermore, imaging studies of patients with schizophrenia performing RL tasks have demonstrated altered activation of fronto-striatal brain networks.^29,53^ The present results suggest that genetic variation in the psychiatric risk gene *CACNA1C* may contribute to the impairments in cognitive flexibility seen in these disorders.

### Alterations in Cacna1c in Rats Impact RL

In a touchscreen reward-based RL task, we found that *Cacna1c* heterozygous knockout rats demonstrated a selective impairment in the early stage of RL. Specifically, fewer *Cacna1c* heterozygous animals successfully reached criteria for reversal. Furthermore, heterozygous animals responded more quickly and with more errors, indicative of behavioral inflexibility and inappropriate responding. This pattern of responses is consistent with a lack of inhibition of previous responding. There was no difference between *Cacna1c* heterozygous animals and controls during the initial visual discrimination component of the task, indicating that these animals could successfully acquire novel stimulus–reward associations. These results therefore suggest that *Cacna1c* heterozygous animals have a selective impairment in inhibition and the switching of reinforced contingencies.

These results in rats show a striking degree of convergence with the effects seen on RL in human risk allele carriers, suggesting that the low-dosage *Cacna1c* rat may represent a valuable model of the effects of genetic variation in *CACNA1C* on cognition in humans. It is notable that some, although not all, reports of the effects of *CACNA1C* genotype on expression suggest that the common variants in humans may be associated with decreased *CACNA1C* expression.^9,10^ Furthermore deleterious mutations in L-type VGCCs have also been associated with risk for schizophrenia in exome-sequencing studies, further implicating low dosage of L-type VGCCs in risk for this disorder.^13^

Our results are broadly consistent with previous studies on mice with forebrain-specific total knockout of *Cacna1c*.^54,55^ Koppe et al^55^ found forebrain-specific conditional *Cacna1c* knockout mice adopt different behavioral strategies during an operant reward-based learning task compared to controls. *Cacna1c* knockout mice did not show an overall behavioral deficit; however, detailed behavioral analysis showed that these animals gained reward based on an outcome-based strategy, rather than learning cue–reward associations, basing their responses more on the previous location of the reward.^55^ Similarly, Temme et al^54^ found that mice lacking forebrain *Cacna1c* could learn contextual and spatial associations normally but were impaired on more subtle tests of context discrimination and pattern completion. A recent study also demonstrated impacts of *Cacna1c* hemizygosity on social behavior and communication.^56^ Our results extend these previous findings to show an impact of genetic manipulation of *Cacna1c* on behavioral flexibility as assessed by RL. In addition, we show a significant behavioral effect of reduced dosage, rather than total ablation, of *Cacna1c*. This is important as genetic variants associated with increased risk for psychiatric disorders in humans are anticipated to affect *Cacna1c* dosage and not to produce a total ablation of the gene (which is not compatible with viability).^9,10,13^

### Impact of Genetic Variation in *CACNA1C* on Prefrontal BDNF Across Species

L-type VGCCs are known to play a critical role in the regulation of BDNF expression.^39,57^ Previous studies in mice completely lacking forebrain *Cacna1c* have also demonstrated a reduction in central BDNF levels.^58^ Furthermore, activity-dependent regulation of BDNF has been shown in rodent models to be critical for both plasticity in the PFC and RL.^35,36^ These findings suggest that altered regulation of BDNF in the PFC may contribute to deficits in RL.

We found that *Cacna1c* heterozygous rats have decreased expression of *Bdnf* in the PFC, a result we confirmed with both qPCR and in situ hybridization. These findings demonstrate that decreased dosage of *Cacna1c* is sufficient to have a significant impact on prefrontal *Bdnf* expression. In addition, we found that *CACNA1C* risk-associated genetic variation in humans is associated with decreased BDNF expression in human PFC, as assessed by selected markers of BDNF in postmortem tissue in the BRAINEAC database. Notably, this effect was not seen for all markers across the *BDNF* gene, suggesting that genetic variation in *CACNA1C* may affect specific BDNF transcripts in the PFC, an area that will be important for future investigation. These results suggest a mechanism through which genetic variation in *CACNA1C* may affect cognitive flexibility and RL, because previous studies have shown that mice lacking activity-regulated expression of *Bdnf* show impaired RL and altered spike-timing-dependent plasticity in the PFC.^35,36^ These results also suggest that therapeutic approaches aimed at enhancing prefrontal BDNF levels may be efficacious in alleviating some of the cognitive impairments seen in neuropsychiatric disorders.

## Supplementary Material

Supplementary material is available at *Schizophrenia Bulletin* online.

sby146_suppl_Supplementary_Material
