# Supplementary material for: Genetic Variation in the Psychiatric Risk Gene CACNA1C Modulates Reversal Learning Across Species
Source: Schizophr Bull. 2018 Oct 10;45(5):1024–32. doi: 10.1093/schbul/sby146 (PMC6737471; doi:10.1093/schbul/sby146)
Supplement: sby146_suppl_Supplementary_Material [file sby146_suppl_supplementary_material.docx]

## SUPPLEMENTARY MATERIAL

### Basic molecular characterisation of Cacna1c hemizygous rats

#### Basal characterisation of Cacna1c expression levels in hemizygous rats

Using *in situ* hybridisation (as per methods), HET animals showed an average 48% reduction of *Cacna1c* mRNA in the hippocampus (t(8) = 4.945, P = 0.001), 37% reduction in the cerebellum (t(6.003) = 3.117, P = 0.021) and 17% in the PFC (t(8) = 2.290, P = 0.051). This was supported by RT-qPCR, which showed a 48% reduction in the CA1 region of the hippocampus (t(18) = 2.933, P = 0.008) and 35% reduction in the PFC (t(26) = 2.963, P = 0.006).

#### Basal characterisation of Cav 1.2 α1 subunit protein expression levels by Western Blotting

We recognise that all currently available Cav 1.2 α1 subunit (Cacna1c) antibodies show some degree of non-specific binding. We therefore conducted independent western blot (WB) analyses, using separate antibodies, in two cohorts of naïve (i.e. non-behaviourally trained) rats to establish Cav 1.2 α1 subunit (Cacna1c) protein expression. We also additionally conducted separate ELISA assays (see below).

The first WB analysis was conducted on hippocampal tissue from WT (n=7) and HET (n=6) animals (Supplementary Figure 1A). Each tissue sample was lysed in RIPA lysis and extraction buffer (Thermo Fisher, UK) with mini protease inhibitor cocktail (Roche Diagnostics) and phosphatase inhibitor (Cell signalling, UK) according to description from manufacturer. After using BCA Assay kit to measure the total amount of protein in each sample, electrophoresis and blotting were carried out with the Mini POTEAN system (BioRad). Gels (4–20% Mini-PROTEAN TGX Stain-Free Gel, 15 well, 15 μl) were run for ∼45 mins at a constant voltage of 150 V and transferred to 0.2μm nitrocellulose blots at 25V, 30min. Blots were blocked in 5% ECL at room temperature (RT) for one hour. Primary antibodies to Ca_v_1.2 α1 subunit (encoded by Cacna1c) (1:500, Rabbit Polyclonal, Alomone, Cat ACC-003) and GAPDH (1:5000, Mouse Abcam) were added in 3% ECL (enhanced luminol-based chemiluminescent) and incubated at 4°C overnight with constant shaking. After three washes with TBST (Tris Buffered Saline with Tween 20), blots were incubated for 1 hour at RT in 3% ECL with secondary antibodies: Goat anti Rabbit 680 RD (1:15000, Li-COR, Cat 926- 68071) for CACNA1C and Donkey anti mouse 800 CW (1:15000, Li-COR, Cat 926-32212) for GAPDH detection.

A second separate WB analysis (Supplementary Figure 1B) was conducted on prefrontal cortical (PFC) tissue from a separate cohort of WT (n = 6) and HET (n = 6) naïve rats. Electrophoresis and blotting were carried out using 4-12% Bis-Tris protein gels (Fisher Scientific, UK). After the electrophoresis, proteins were transferred onto nitrocellulose membrane (GE Healthcare, UK). The membrane was blocked in 3% BSA in 0.2% TBST for one hour at the room temperature and incubated in a different Ca_v_1.2 α1 subunit primary antibodies overnight at 4^o^C (anti-Ca_v_1.2 primary antibody AB5156, 1:200, EMD Millipore). GAPDH still used as loading control (1:5000, from Abcam). Fluorescent secondary antibodies (Goat anti Rabbit 680 RD and Goat anti Mouse 680 RD, 1:15,000, Li-Core, UK) were used to detect Ca_v_1.2 α1 subunit and GAPDH protein level. All Blots were washed in TBST, imaged and quantified using Odyssey CLX imaging System (LI-COR Biosciences). Measurements were taken from three digital images with different exposure times that optimised signal resolution above background in the pseudolinear range for bands at 250 kDa, ~150 kDa and GPAPH.

Overall we observed a significant reduction of Ca_v_1.2 α1 subunit protein (Cacna1c) in the hippocampus in HET compared to WT rats (Supplementary Figure 1A) and also a significant reduction in the PFC in HET rats (Supplementary Figure 1B) with the two antibodies. There was no difference between WT and HET in the non-specific ~ 150KDa protein band in either assay^1^. We acknowledge that in addition to the lack of change in the non-specific band it would be ideal to further test these antibodies using tissue from a full knockout model to confirm loss of specific binding. Unfortunately Cacna1c homozygous knockout rats are not viable and therefore this assay could not be conducted for rat tissue in the current project, but this remains a future objective.

#### Basal characterisation of Ca_v_1.2 α1 subunit protein expression levels by ELISA

BlueGene ELISA kits (American Research Products Ltd) were used to quantify Ca_v_1.2 α1 subunit protein (encoded by *Cacna1c*) using the competitive enzyme technique for PFC, cerebellum and hippocampus. Experiments were conducted as per manufacturer’s protocols. All kit components were brought to room temperature before use. Samples were diluted 1:2 in extraction buffer. 100 μl of standards, samples and T-PER control were added to wells with an additional 10 μl of supplied balance solution added to the samples and one set of control wells. 50 μl of HRP enzyme conjugate was added to each well, excluding the control wells, and the plate was covered and incubated at 37 ⁰C for 1 hour. The plate was washed manually 5 x with 1 x wash buffer, with all liquid aliquoted after each and blotted dry on clean paper towel following the final wash. The plate was covered and incubated for 15 mins at 37 ⁰C with a substrate for HRP enzyme, with 50 μl of supplied substrate A and substrate B added to each well. A stop solution was added (50 μl) and plates were evaluated within 5 mins. Plates were evaluated using a CLARIOstar microplate reader (BMG LABTECH Ltd, Bucks, UK) set at 450 nm and 550 nm. For analysis, 550 nm values were extracted from 450 nm values to account for any optical imperfections in the plate. Six standards were run alongside to provide a standard curve for quantification, as well as a blank control. A standard curve was produced from the standards, with the equation of the line used to calculate the concentration for each sample. Values were multiplied by dilution ratio and normalised to sample protein concentration. Ca_v_1.2 α1 subunit (Cacna1c) protein levels were significantly reduced by 48% in the cerebellum and 31% in the PFC (P = 0.020 and P = 0.041, respectively) (raw plate reader output values – Cerebellum WT (n=8) 0.72 (SEM 0.08), HET (n=8) 0.38 (SEM 0.09); PFC WT (n=8) 0.74 (SEM 0.07), HET 0.51 (n=8) (0.08)). No compensatory changes were observed in Cacna1d (Ca_v_1.3 α1 subunit) in the same brain regions.

qPCR methods

Using the QIAGEN RNeasy kit, RNA was isolated from prefrontal cortex and hippocampal tissue from individual animals (11 WT and 10 HET). Samples were DNAase treated using TURBO DNA-free TM  Kit (Ambion Life Technologies), following the recommended protocol. cDNA synthesis was performed using the RNA to cDNA EcoDry TM  Premix (Random Hexamers) synthesis tubes (Clontech), heated at 42°C for 75 minutes, followed by 80°C for 15 minutes. qPCR was conducted with SensiMix SYBR Green (Bioline) on the StepOne Plus (Life Technologies; 1 cycle 95°C, 10 mins; 45 cycles of 95°C, 15 secs and 60°C, 1 min; with melt curves conducted 55°C, 1 min; 95°C for 15 secs). All qPCR samples were run in triplicate and the outcome was calculated using 2 ΔΔCt method, normalised to UBC and SDHA housekeeping genes. Primer sequences are given in Supplementary Table 2.

### Basic behavioural characterisation of Cacna1c hemizygous rats

### Animals

Thirty-two male Sprague Dawley rats (16 WT and 16 HET litter mates) were housed in groups of 2-4.

### Methods

###### *Locomotor activity*

Activity levels were tested in empty home-cage equivalent contexts for 2 hrs on 3 consecutive days. The number of beam breaks and the number of runs were counted for each animal, split into four 30 min bins. Total beam breaks and runs were compared between WT and HET animals as measures of locomotor activity, using independent samples student’s t-tests. Differences within the sessions and across the 3 testing days were compared within animals and between genotype using repeated measures ANOVA, as a measure of habituation to a novel context.

*Open field exploration*

Animals were placed into an arena (1 m^2^) for 10 mins. A central zone was defined as the centre 70 cm x 70 cm square. Distance and speed travelled were recorded for each animal. Duration of time spent in the central zone was recorded, with greater time spent in the central zone indicating reduced anxiety. Student’s t-tests were used to compare distance, speed and time in the central zone between WT and HET animals.

###### *Startle response*

Animals underwent a 30-min startle response session in a SR-Lab^TM^ Startle Response System, consisting of 91 trials. Background noise level was set to 70 dB and test pulses were administered at 120 dB and 105 dB. At the end of the session there were 6 additional pulses of increasing intensity; 70-120 dB to measure increasing startle response. An accelerometer sensor measured the amount of movement of each animal in response to each test pulse. Repeated measures ANOVA was used to analyse the effect of increased pulse intensity and genotype on startle response. Student’s t-tests were used to compare overall startle response for 120 dB pulses alone and 105 dB pulses between WT and HET animals.

### Results

###### *Locomotor activity*

There was no difference in the total number of beam breaks or runs made between WT and HET animals across the full 3 days of testing (t(29) = -1.666, P = 0.107 and t(29) = -1.526, P = 0.138 respectively). All animals showed normal habituation to context exposure within session, with fewer beam breaks and runs being made in the last half hour of the sessions compared to the first (F(2.069, 59.990)_Ɛ = 0.690_ = 184.494, P < 0.001). All animals showed between session habituation with fewer breaks and runs observed on testing day 3 compared to day 1. Repeated measures ANOVA reported a significant effect of day on beam breaks and runs (F(2,58) = 5.465, P = 0.007, and F(2,58) = 5.695, P = 0.006, respectively). There was no evidence of an interaction between day and genotype for either beam breaks or runs (F(2,58) = 0.169, P = 0.845 and F(2,58) = 0.543, P = 0.584 respectively).

###### *Open Field Exploration*

There was no difference in activity levels between WT and HET animals, with similar distance travelled and similar mean and max velocities during the task (t(30) = -0.294, P = 0.771; t(30) = -0.293, P = 0.771; and t(30) = -0.064, P = 0.950 respectively). WT and HET animals did not show significant difference in the amount of time spent in the central zone during the 10 min exploration (t(30) = 1.567, P = 0.128).

###### *Startle response*

There was no effect of genotype on startle to 120 dB or 105 dB test pulses compared to 70 dB background noise (t(29) = 1.070, P = 0.293 and t(29) = -0.240, P = 0.813 respectively). Increasing test pulses in increments of 10 dB, from 70 dB to 120 dB, resulted in the expected increase in startle response (F(1.444, 41.872) _Ɛ = 0.298_ = 145.237, P < 0.001), with no effect of genotype (F(1,29) = 0.987, P = 0.329)

#### ***Quantitative* in situ *hybridisation methods***

*In situ* hybridisation was used to quantify the expression of *Bdnf* and *Cacna1c* in rodent tissue using established techniques^2^. In brief 14µm coronal slices of the PFC, cerebellum and hippocampus were taken from ten whole brain samples (5 HET and 5 WT) mounted on poly-L-lysine coated slides. Slides were fixed with 4% PFA, dehydrated and stored in 95% ethanol until use. Probes for *Cacna1c* (3’ - TCGAAGTAGGTGGAGTTGACCACGTACCACACTTTGTACTGGTGC – 5’) and *Bdnf* exon IX (3’ – CGAACCTTCTGGTCCTCATCCAGCAGCTCTTCGATCACGTGCTCA – 5’) were radiolabelled using deoxyadenosine 5’- (α-thio) triphosphate [^35^S] (dATP) (Perkin Elmer). Hybridisation was conducted as previously described. To define non-specific hybridization, adjacent slide-mounted sections were incubated with radiolabeled oligonucleotide in the presence of an excess (80X) concentration of unlabelled oligonucleotide probe. Slides were apposed to Carestream Biomax MR film (Anachem) and exposed for 1 week before being developed. Image analysis was conducted using ImageJ software, with optical density values quantified using a ^14^C ladder. Ten measurements were made for each region of interest from each brain slice, to include the prefrontal cortex (PFC, averaged across the ventromedial, infralimbic and paralimbic), hippocampus and cerebellum. A specific hybridization signal was calculated for each region of interest by calculating the difference between mean total and non-specific values for an individual rat. Specific hybridisation values were then averaged within genotype and normalised to mean WT by region.

### CACNA1C and Reversal Learning in Humans

### Participant Characteristics

### Participants had no history of psychiatric illness (themselves or first-degree relative) and did not report taking any psychotropic medication or illegal substances. All participants were right handed university graduates (with 17+ year’s education). A total of 84 participants (mean age 23.95 ± 3.64 s.d, 49 female) were included in the analysis, after exclusion for quality control of genetic data (N=10) or incomplete/missing behavioural data (N=6) .

### Reversal learning procedure in human participants

Participants learned to choose one of two simultaneously presented colours (‘blue’ and ‘green‘) by receiving monetary reward for correct choices and monetary punishment for wrong choices (e.g. +1 pence [p] for ‘blue’ and -1p for ‘green‘). After 7-11 trials, reward/punishment contingencies were reversed so that the previously rewarded colour was now punished and vice versa. Participants were instructed to maximize their earnings during the learning session, which consisted of 12 reversal episodes in total (108 choice trials). Within each reversal episode we included either 1 or 2 PE (probabilistic error) trials, in which ‘wrong’-feedback was given for correct choices, even though the reward contingencies had not changed. At the start of each choice trial, participants were presented with a response cue consisting of two white frames surrounding the colours and prompting the participants to press the left or right button on a response box to choose one colour. Response feedback (choice outcome) was given subsequently using a centrally presented white ‘smiley’ (correct choice) or red ‘frowny’ (incorrect choice) face and an earnings counter changing incrementally by +/- 1p. Overall learning performance was assessed as the accumulated earnings across all 108 choice trials. We also calculated trial-based average accuracies (% choices corresponding to the correct colour of each reversal episode) for the trials directly following reversal events (post-reversal trials) for each participant.

### Reversal learning – CACNA1C genotype effects

For each individual, we additionally calculated the percentage of correct responses for each trial in a 7-trial window around a reward reversal. For each of the seven trials, we ran an analysis of covariance for main effects focussing on the rs2007044 genotype, controlling for age, gender and composition cognition, as measured via CompP from MATRICS MCCB^3^. Consistent with our observations in the Cacna1c heterozygous rat model, the genotype effect was specific to trials immediately following a reversal of reward contingency (highlighted in grey) and to a weaker extent in the second trial following reversal (Supplementary Figure 2 and Supplementary Table 3). We did not observe any effect of genotype on reaction time (P > 0.1 in all cases).

We further assessed trial-by-trial performance of post-reversal accuracy across genotype, focussing on the rs2007044 SNP (across the individual 12 post-reversal trials, per subject). In a random mixed effects–model, we find evidence of an additional rs2007044 genotype × trial number interaction (adjusted: F_2,800_ = 2.95, P = 0.053; above and beyond the main effect of rs2007044 genotype), providing some preliminary evidence that the homozygous non-risk allele group learned to perform in post-reversal trials better towards the end of the task, compared to G risk allele carriers.

(A)


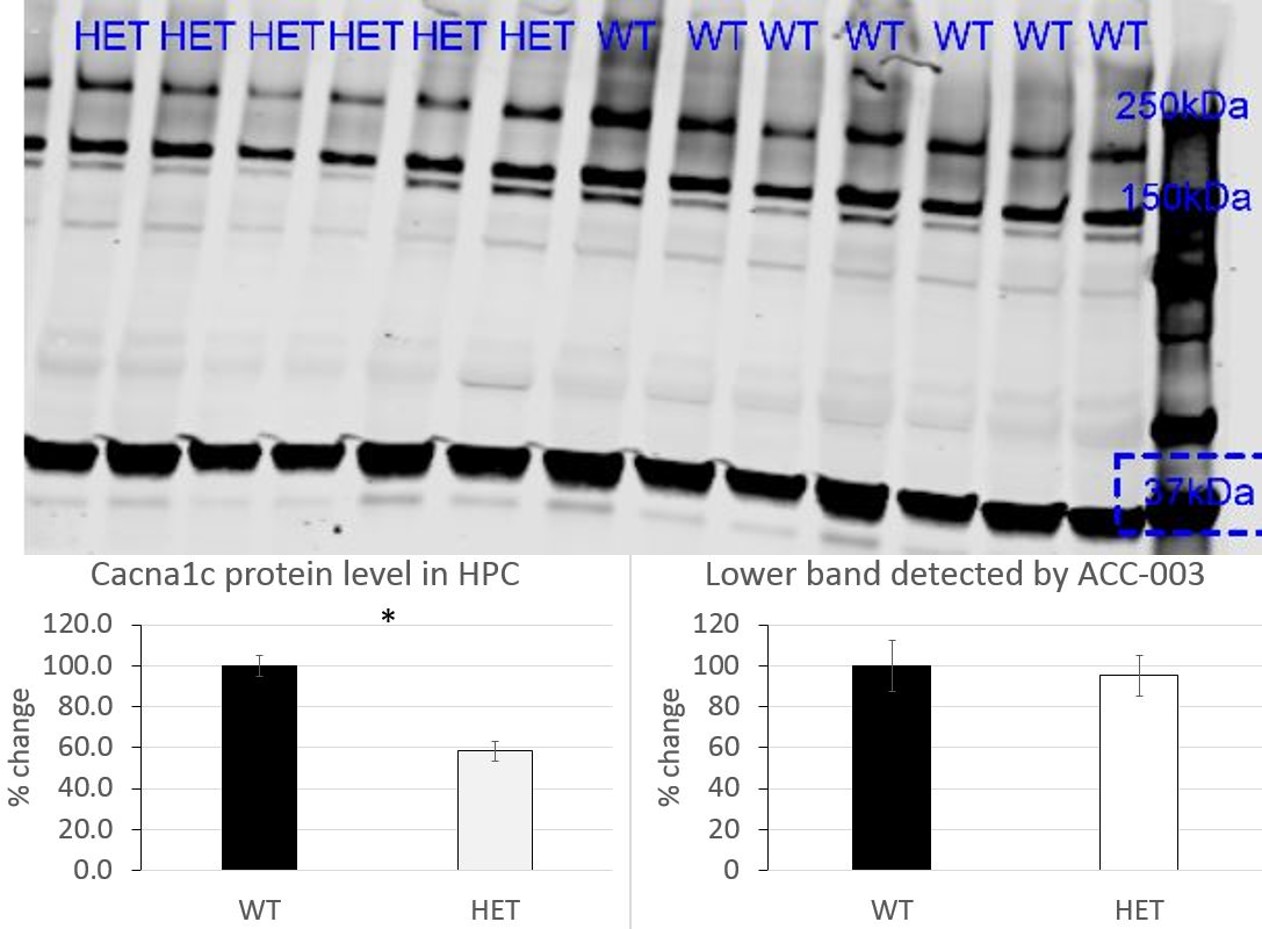


(B)


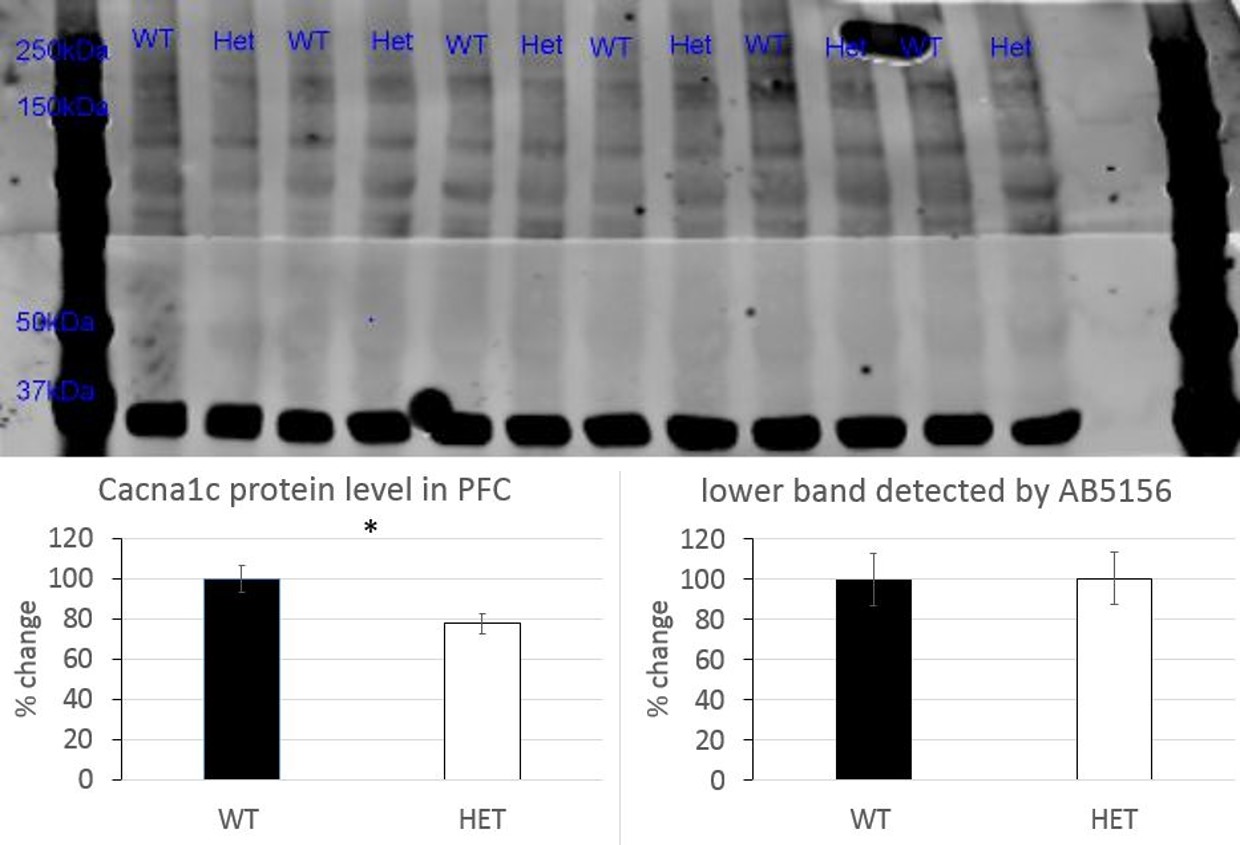


**Supplementary Figure 1**. Western blot for Ca_v_1.2 α1 and GAPDH in heterozygous (Het) and wild type (WT) animals showing a 40% reduction in Ca_v_1.2 α1 protein level in the hippocampus (A) and a 23 % reduction PFC (B). The results were confirmed in two independent analyses of the samples with each antibody. n = 6 all groups except WT in A (n = 7). * P < 0.05 compared to WT, ANOVA.

***
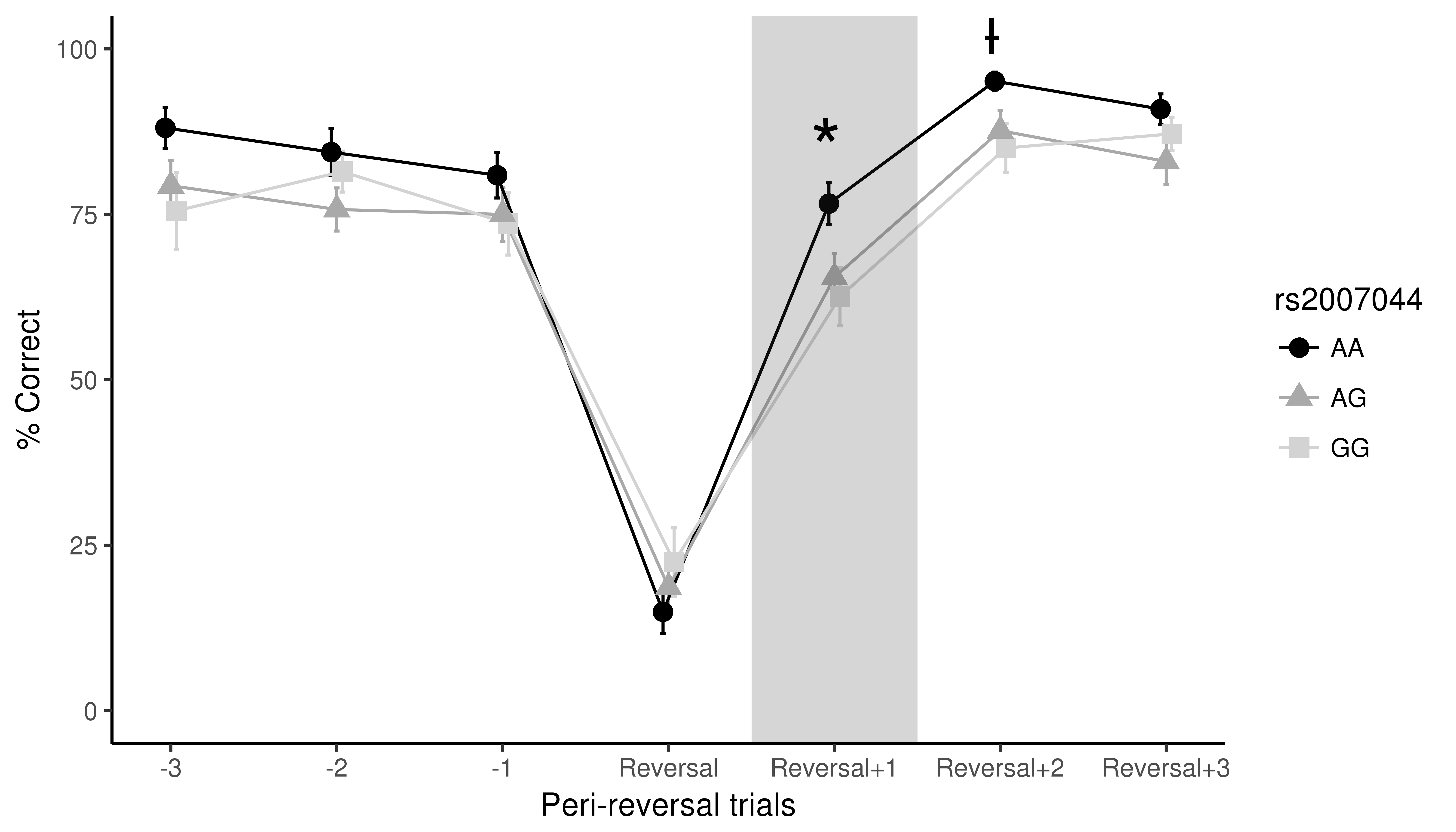
***

**Supplementary Figure 2**. Proportion of correct responses within peri-reversal window, stratified by rs2007044 genotype (mean ± standard error).* P < 0.05; Ɨ P < 0.08, correcting for age, gender and composite cognition. Grey bar represents post reversal trials.


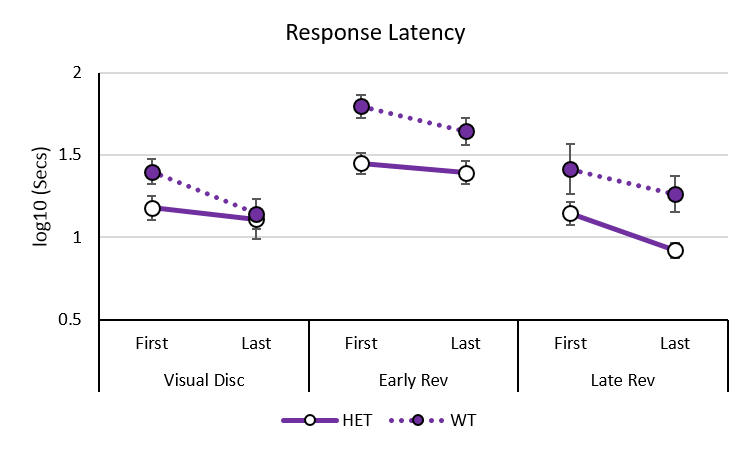


**Supplementary Figure 3**. Response latencies for WT and HET animals compared at start and end of "Visual discrimination", "Early" and "Late" reversal. Latencies are averaged across the first 3 and last 3 sessions of each phase respectively. Error bars are SEM. Significantly faster responding is seen in HET animals during “Early” reversal, P < 0.05.

**Supplementary Figure 4.** Data derived from BRAINEAC ([www.braineac.org](http://www.braineac.org)) showing that CACNA1C risk allele carriers at rs1006737 and rs2007044 show decreased expression of BDNF in the frontal cortex with probe set exprID 336723 (P=0.007 and P=0.042 respectively).

| **Experimental Condition** | **Description** | **Criterion to move onto next phase** |
| --- | --- | --- |
| Habituation | Habituation sessions required animals to collect a sucrose solution reward from the magazine, then initiate the next trial by exiting the magazine. | Completing a minimum of 100 trials in the 30 mins session on 2 consecutive days of training. |
| Must Touch | Stimuli are displayed randomly on the screen one at a time. (Stimuli consist of various shapes. One stimulus is presented at a time, on one side of the screen. The other side of the screen is left blank. The left or right position is chosen pseudo randomly, such that any image will not be displayed on the same side more than 3 times in a row.) The rat *must touch* the stimulus to elicit the liquid reward. There is no response if rat touches the blank | Completing a minimum of 100 trials with fewer than 20 blank touches on 2 consecutive days of training. |
| Visual Discrimination | Two stimuli (S+ vs S-) are presented at a time, on either side of the screen. (The stimulus position is chosen pseudo randomly). The rat must touch the correct stimulus (S+) to acquire the liquid reward. Touching the incorrect stimulus (S-) terminates the trial and the houselight is turned on for a time-out period of 10s and no reward is given. | Respond at greater than 80% correct a session on 2 consecutive days of training.  % correct = (trials to which a correct response was made/total trials) *100 |
| Early reversal | As Visual Discrimination but the contingencies are reversed. | Return to 50% correct responding within the session after the contingencies are reversed. |
| Late reversal | As Early Reversal | Respond at greater or equal to 80% correct within a session on 2 consecutive days of training with the reversed contingencies. |

**Supplementary Table 1** Description and criteria for advancing from each experimental condition in the animal reversal learning paradigm.

| **Gene** | **FWD 5’-** | **REV 5’ -** |
| --- | --- | --- |
| CACNA1C | ATGGTTCTTGTCAGCATGTTGCGG | TGCAAATGTGGAACCGGTGAAGTG |
| BDNF IX | GGTTATTTCATACTTCGGTTGC | CCCATTCACGCTCTCCAG |
| UBC | CTTTGTGAAGACCCTGAC | CCTTCTGGATGTTGTAGTC |
| SDHA | GCTCTTTCCTACCCGCTCAC | GTGTCATAGAAATGCCATCTCCAG |

**Supplementary Table 2**. Primer sequences used in qPCR.

|  | rs2007044 genotype | | | | | | Adjusted Model | |
| --- | --- | --- | --- | --- | --- | --- | --- | --- |
|  | AA  N=28 | | AG  N=39 | | GG  N=17 | | F_2,81_ / χ^2^ | P |
|  |  |  |  |  |  |  |  |  |
| Age | 24.46 | 5.56 | 24.21 | 4.09 | 23.88 | 2.93 | 0.091 | 0.913 |
| Sex (M / F) | 10 | 18 | 21 | 18 | 3 | 14 | 6.8345 | 0.033 |
| MCCB CompP | 58.38 | 23.4 | 62.11 | 23.87 | 64.31 | 22.81 | 0.361 | 0.698 |
|  |  |  |  |  |  |  |  |  |
| -3 Reversal | 88.07 | 16.53 | 79.34 | 23.98 | 75.54 | 23.91 | 2.402 | 0.097 |
| -2 Reversal | 84.41 | 18.75 | 75.74 | 20.35 | 81.46 | 12.74 | 2.014 | 0.140 |
| -1 Reversal | 80.91 | 18.21 | 74.99 | 25.25 | 73.6 | 19.58 | 0.934 | 0.397 |
| Reversal | 14.94 | 17.18 | 18.65 | 19.67 | 22.46 | 21.35 | 0.845 | 0.433 |
| +1 Reversal | 76.62 | 16.68 | 65.5 | 22.29 | 62.57 | 18.15 | **3.123** | **0.049** |
| +2 Reversal | 95.13 | 7.21 | 87.65 | 18.83 | 85.03 | 15.39 | 2.614 | 0.079 |
| +3 Reversal | 90.91 | 12.12 | 82.98 | 21.89 | 87.17 | 10.19 | 2.003 | 0.142 |
|  |  |  |  |  |  |  |  |  |

**Supplementary Table 3**. Demographic / behavioural summary data for rs2007004 in human subjects (mean ± sd). For adjusted models, all rs20070044 effects on reversal learning behavior are corrected for age, gender and composite cognition (as measured via CompP from MATRICS MCCB^3^).

| ***Sessions to criteria (Mean (range))*** | |  |
| --- | --- | --- |
|  | WT | Het |
| Habituation | 3.5 (3-4) | 3.3 (3-4) |
| Must Touch | 15.3 (11-37) | 21.2 (10-44) |
| Visual Discrimination | 24.4 (11-44) | 27.8 (3-71) |
| Early Reversal | 18.5 (8-33) | 16.8 (10-40) |
| Late Reversal | 32.3 (8-57) | 16.75 (6-31) |
| Full (Early + Late) Reversal | 50.3 (28-70) | 30.7 (16 -53) |
| ***Trials to criteria (Mean (Range))*** | |  |
|  | WT | Het |
| Habituation | 256.3 (200-362) | 236.4 (155-380) |
| Must Touch | 755.8 (277-1874) | 741.5 (372-1408) |
| VD | 949.3 (560-1475) | 833.5 (273-1499) |
| Early Reversal | 272.1 (63-751) | 337 (128 - 570) |
| Late Reversal | 848.6 (282 -1204) | 761.2 (390 -1430) |
| Full (Early + Late) Reversal | 1146.1 (450 - 1503) | 1137 (695-1727) |

**Supplementary Table 4**. Mean number of session and trials (with range) to reach criteria for WT and HET animals that completed each stage.

***Supplementary References***

1. Buonarati OR, Henderson PB, Murphy GG, Horne MC, Hell JW. [Proteolytic processing of the L-type Ca _2+_ channel alpha _1_1.2 subunit in neurons.](https://www.ncbi.nlm.nih.gov/pubmed/28781760) F1000Res. 2017 Jul 21;6:1166. doi: 10.12688/f1000research.11808.1. eCollection 2017.

2. Wisden BM. In Situ Hybridization Protocols For the Brain (Biological Techniques). San Diego: Academic Press; 1994

3. Kern RS, Gold JM, Dickinson D, et al. The MCCB impairment profile for schizophrenia outpatients: results from the MATRICS psychometric and standardization study. Schizophr Res Mar 2011;126(1-3):124-131.
